# Prussian Blue Nanoparticles as a Versatile Photothermal Tool

**DOI:** 10.3390/molecules23061414

**Published:** 2018-06-11

**Authors:** Giacomo Dacarro, Angelo Taglietti, Piersandro Pallavicini

**Affiliations:** 1 inLAB—Inorganic Nanochemistry Laboratory, Dipartimento di Chimica, Università di Pavia, 27100 Pavia, Italy; angelo.taglietti@unipv.it; 2 CHT, Centre for Health Technologies, Università di Pavia, 27100 Pavia, Italy

**Keywords:** prussian blue: nanoparticles, photothermal effect, nanomedicine, theranostics

## Abstract

Prussian blue (PB) is a coordination polymer studied since the early 18th century, historically known as a pigment. PB can be prepared in colloidal form with a straightforward synthesis. It has a strong charge-transfer absorption centered at ~700 nm, with a large tail in the Near-IR range. Irradiation of this band results in thermal relaxation and can be exploited to generate a local hyperthermia by irradiating in the so-called bio-transparent Near-IR window. PB nanoparticles are fully biocompatible (PB has already been approved by FDA) and biodegradable, this making them ideal candidates for in vivo use. While papers based on the imaging, drug-delivery and absorbing properties of PB nanoparticles have appeared and have been reviewed in the past decades, a very recent interest is flourishing with the use of PB nanoparticles as photothermal agents in biomedical applications. This review summarizes the syntheses and the optical features of PB nanoparticles in relation to their photothermal use and describes the state of the art of PB nanoparticles as photothermal agents, also in combination with diagnostic techniques.

## 1. Introduction

**Figure 1:**
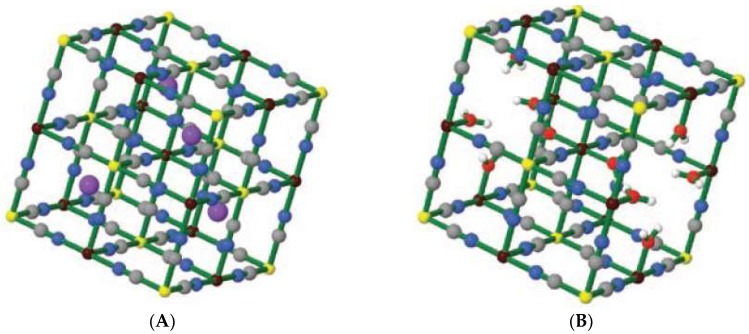
(**A**) Rendering of the lattice of ‘soluble’ PB, KFe^III^[Fe^II^(CN)_6_], with half of the centers of the cubic cells occupied by K^+^. Colors: Fe^II^ yellow, Fe^III^ brown, C gray, N blue, K, violet; (**B**) Rendering of the lattice of ‘insoluble’ PB, Fe^III^_4_[Fe^II^(CN)_6_]_3_, with the coordinative sphere of Fe^III^ completed by water molecules (O red, H white). Reproduced by permission from Ref. [1].

Prussian Blue (PB) is believed to be the oldest artificial coordination compound. It was accidentally prepared in the early 18th century by the colormaker Heinrich Diesbach from Berlin [1], its name following the geopolitical origin. PB is indeed a coordination polymer, containing Fe^3+^ cations and the hexacyanoferrate complex, [Fe(CN)_6_]^2+^, the latter being the well known octahedral complex with the d^6^ Fe^2+^ cation in its low spin state. In PB the Fe^2+^ cations are coordinated by the carbon atom of the cyanide (CN^−^) ligand, that acts as a bridge with Fe^3+^ cations, that are octahedrally coordinated by 6 nitrogen atoms. The preparations of PB typically require mixing of Fe^3+^ salts with potassium hexacyanoferrate, K_4_[Fe(CN)_6_], leading to different formula depending on the exact stoichiometry and preparation conditions. Such formula may range from Fe^III^_4_[Fe^II^(CN)_6_]_3_∙xH_2_O (x = 14–16) and KFe^III^[Fe^II^(CN)_6_]∙xH_2_O (x = 1–5) [1] that are commonly called ‘insoluble PB’ and ‘soluble PB’, respectively (oxidation states as roman superscripts are added for sake of clarity). Another way of formulating PB, neglecting water molecules, is K_x_Fe^III^[Fe^II^(CN)_6_]_(3+x)/4_, with x ranging from 0 (insoluble PB) to 1 (soluble PB) [2]. All these formula corresponds indeed to the same product, as it has been made clear by crystal and molecular structure determination by x-ray diffraction [3]. PB has a cubic lattice with a face centered cubic unit cell alternating Fe^2+^ and Fe^3+^ cations bridged by cyanide. If half of the centers of the cubic cells are occupied by K^+^ the ideal formulation of ‘soluble’ PB is obtained, i.e., KFe^III^[Fe^II^(CN)_6_], with a further low number of water molecules in the lattice, see Figure 1A. On the other hand, as the ideal formulation of ‘insoluble’ PB is Fe^III^_4_[Fe^II^(CN)_6_]_3_, a quarter of the Fe(CN)_6_]^4−^ complexes must be absent and some N-sites around Fe^3+^ are occupied by water molecules [1,2,3], Figure 1B, this explaining the higher number of water molecules found in the ‘insoluble PB’ formula. ‘Soluble’ and ‘insoluble’ PB owe their name to the tendency of the former to give lower dimensioned crystals that reach the size typical of the mesophase, i.e., that exist as nanoparticles, forming clear deep-blue colored colloidal solutions. ‘Insoluble PB’ instead forms larger crystals that easily aggregate and give a precipitate. It has to be stressed that PB can be prepared both by mixing Fe^3+^ with [Fe^II^(CN)_6_]^4−^ (the ferrocyanide anion) and by mixing Fe^2+^ with [Fe^III^(CN)_6_]^3−^ (the ferricyanide anion). In the latter case the obtained blue product historically received a different name, Turnbull Blue, however it is indeed PB [4]: it forms after a redox reaction leading to Fe^3+^ and [Fe^II^(CN)_6_]^4−^, promoted by the coordination of the cyanide N atom to the Fe^2+^ cations, that would be otherwise endoergonic on the basis of standard redox potentials (E°(Fe^3+^/Fe^2+^) = 0.77 V, E°([Fe^III^(CN)_6_]^3−^/[Fe^II^(CN)_6_]^4−^) = 0.37 V [5]).

**Figure 2:**
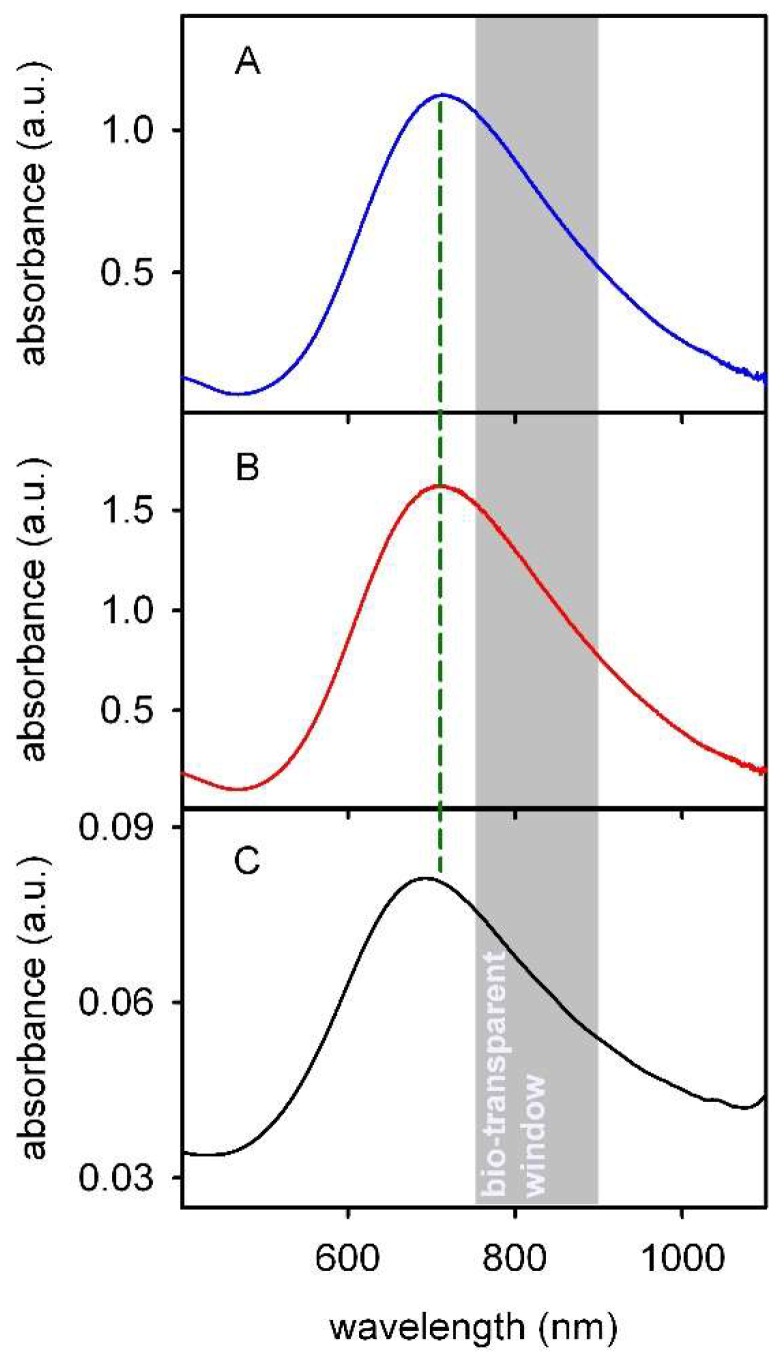
Absorption spectrum of PBnp. (**A**) solution, prepared from Fe^3+^ and [Fe^II^(CN)_6_]^4−^; (**B**) solution, prepared from Fe^2+^ and [Fe^III^(CN)_4_]^3−^; (**C**) dry (air interface) self-assembled monolayer on glass, prepared using PBnp with the absorption spectrum (**A**). The green dotted line evidences the position of the maximum of absorption. The grey area highlights the ‘bio-transparent’ region of the spectrum. (Authors’ graphical rework from data of Ref. [10]).

The blue colour of PB is due to a charge transfer transition, i.e., to the transfer of one electron from the Fe^II^ centers to the Fe^III^ centers [6]. As we have stressed, while Fe^II^ is invariably octahedrally coordinated by 6 C atoms of the cyanide anions, Fe^III^ is octahedrally coordinated by 6 N atoms of the cyanide anions in ‘soluble’ PB and by an average of 4.5 N atoms plus 1.5 oxygen atoms (H_2_O) in the ‘insoluble’ PB. This may explain the subtle differencies in the position of the absorption maximum of PB, that is tipically reported to be at “around 700 nm” [2]: e.g., at 709 nm [6], 712 nm [7], 700 nm [8]. Spectroelectrochemical experiments allowed to determine the spectrum of ‘insoluble’ PB, i.e., Fe^III^_4_[Fe^II^(CN)_6_]_3_, deposited on a SnO_2_ electrode, with λ_max_ at 700 nm and molar extinction coefficient (ε) expressed with respect either to Fe^2+^ (9800 L mol^−1^ cm^−1^) or to Fe^3+^ (7300 L mol^−1^ cm^−1^) [9]. Although the PB maximum of absorption falls in the visible, such huge ε values typical of CT bands and the large FWHM (full width at half maximum) allow efficient absorption also in the NIR, especially in the so-called ‘bio-transparent window’, i.e., the 750–900 nm range [10], see Figure 2. Relaxation of excited PB is non-radiative but thermal, resulting in local temperature increase after NIR irradiation. Moreover, PB has an excellent biocompatibility and it has been approved by the US FDA (Food and Drug Administration) thanks to its in vivo stability and ability to sequestrate Cs^+^ and Tl^+^ from water and from the human body, allowing their excretion [11,12,13,14,15]: Radiogardase^®^ is a commercial drug made of ‘soluble’ PB (i.e., PB nanoparticles), used to treat patients after Tl intoxication or ^137^Cs contamination. The easy synthesis, the long term stability in a wide range of conditions, the biocompatibility supported by FDA approval and the photothermal response on NIR irradiation has led to a recent burst in the interest towards biomedical applications of PB nanoparticles (PBnp) in PTT (photothermal therapy). This area has not yet been specifically reviewed and the present paper proposes to fill such gap. On the other hand different biomedical uses of PBnp have been reported, especially in the field of imaging [16,17,18,19] and drug delivery [16,20,21,22,23], that will not be reviewed in this paper. Finally, at this stage the so-called Prussian Blue analogs (PBA) have to be mentioned. These are coordination polymers with the general formula A_x_M_y_[M’(CN)_6_]_z_, where A is an akaline metal cation, and M and M’ are metal cations in oxidation state +2 or +3.

Due to their rich set of properties and potential technological and biomedical appplications, a huge number of papers has recently appeared on PBA materials at the nanoscale. In many cases their absorption spectrum has CT bands of similar intensity as PB, falling however in the visibile, i.e., out of the bio-transparent window, this seriously limiting their possible use as PTT agents. Due to this, PBA-based nanomaterials will not be included in this review, except where relevant for the comparison with the photothermal properties of PBnp. We address the reader interested in the properties and biomedical applications of PBA nanoparticles to recent dedicated reviews [2,24,25].

## 2. Synthesis

### 2.1. Homogeneous PBnp

**Figure 3:**
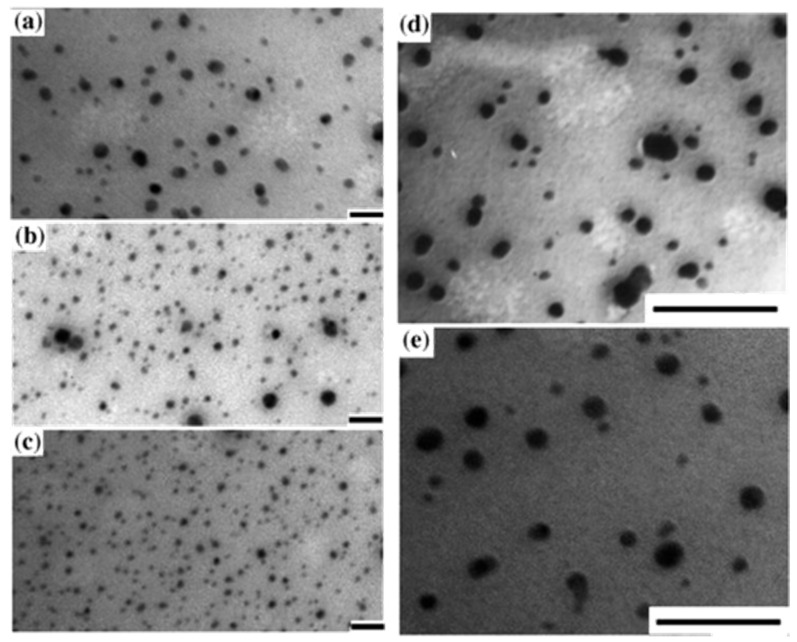
TEM micrograph of size-tunable PVP-coated spherical PBnp from Reference [27]. Nanoparticles were prepared with [Fe^2+^] = [Fe^3+^] = 10 mM and different feed ratios of PVP/Fe^2+^ = (**a**) 20, (**b**) 50, and (**c**) 100, respectively. PB nanoparticles (**d**) prepared at [Fe^2+^] = [Fe^3+^] = 1 mM, PVP/Fe^2+^ = 100, and (e) obtained from the redispersed sample in CHCl_3_ (average size of the original particles was 16 nm). Scale bars = 100 nm. Reprinted with permission from *J. Am. Chem. Soc.* **2003**, *125*, 7814–7815. Copyright 2003 American Chemical Society.

Although PB is known since the 18th century also in its ‘soluble’ (i.e., nanosized) form, the rationalization of PBnp synthesis, with control in size and shape, dates only to the new millenium. Cubic PBnp with 12–16 nm length were prepared for the first time by confining their synthesis in a nanoscaled volume, i.e., in water droplets formed in reverse microemulsions obtained from the anionic surfactant AOT (sodium bis(2-ethylhexyl)sulfosuccinate) in isooctane [26]. The synthesis proceeded through a photochemical route, by the slow photoreduction of [Fe^III^(C_2_O_4_)_3_]^2−^ to Fe^2+^, that in turn reacted with (NH_4_)_3_[Fe^III^(CN)_6_] to generate (NH_4_)_x_Fe^III^_y_[Fe^II^(CN)_6_] nanoparticles (x + 3y = 4). These PBnp are hydrophobic due to the adhesion of the AOT sulfate head to their surface. These PB nanocubes tend to form 2D superlattices when dropcasted on grids for TEM imaging, mainly due to the solvent evaporation and consequent hydrophobic interaction between their AOT coating. Their absorption spectrum was reported to have a maximum at 680 nm. Less regular, spheroidal PBnp were obtained in 2003 by the aqueous reaction of Fe^2+^ with [Fe^III^(CN)_6_]^3−^ in the presence of polyvinylpyrrolidone (PVP), that limited the growth and also acted as the protective coating [27]. The dimensions of these PBnp was tuned between 12 and 25 nm by the iron reactants concentration (see Figure 3). Such PBnp were also obtained as a powder, that it was possible to redissolve in a range of organic solvents thanks to the amphiphilic PVP nature. Interestingly, the absorption spectrum maximum of the PVP-coated PBnp slightly changed with the solvent polarity, as expected for CT bands, ranging from 672 nm in CHCl_3_ to 689 nm in water. It has to be noted that despite the hugely different dielectric constant of water (80.4 at 20 °C) and CHCl_3_ (4.8 at 20 °C) only a 3 nm shift was observed. This is due to the continuous nature of PBnp, in which most of the Fe^III^-NC-Fe^II^ moieties responsible for the CT absorption are buried in the bulk of the nanoparticles and not in direct contact with the solvent. Moreover, also the PVP coating (thickness not given in the paper) is expected to minimize the variation of local dielectric constant for the nanoparticles, resulting in a scarce dependence of λ_max_ from the environment.

PVP-protected spheroidal PBnp were also synthesized in isooctane/AOT microemulsions, with the use of compressed CO_2_ as the antisolvent to recover the coated nanoparticles from the solution [28]. Separate microemulsions containing FeCl_2_ and K_3_Fe(CN)_6_ were prepared using aqueous solutions with 1.5% PVP and then mixed to obtain the product. Tuning the water/AOT molar ratio allowed to regulate the average PBnp dimensions between 20 and 27 nm. In all cases, the colloidal dispersions of these PBnp displayed the usual CT band with λ_max_ = 690 nm. The segregation approach was also used in an elegant synthesis of ferritin-protected PBnp [29]. First, apoferritin was dissociated into subunits at pH 2, followed by its reconstruction at pH 8.5 in the presence of [Fe^III^(CN)_6_]^3−^, giving rise to a solution containing ferritin-trapped [Fe^III^(CN)_6_]^3−^ (untrapped hexacyanoferrate(III) was eliminated from solution by prolonged dialysis). Addition of Fe^2+^ lead to the formation of small, spherical PBnp (<5 nm), whose shape and dimensions were obviously driven by the shape and internal dimensions of the ferritin container. The λ_max_ of the ferritin-PBnp was 710 nm.

The use of a stabilizing polymer or cage was for long the only route followed to synthetize PBnp: several “green” syntheses and molecules of biological origin, such as chitosan [30], soluble starch [31] and viral capsids [32].

PBnp wihtout any stabilizing polymer can be obtained simply by mixing FeCl_3_ and K_3_Fe(CN)_6_ in presence of H_2_O_2_ [33]. The use of sonochemistry in this kind of synthesis helped obtaining smaller nanoparticles (i.e., 5 nm in size instead of a size of ≈50 nm obtained without sonication) [34]. A synthetic route based on [Fe(CN)_6_]^4−^ as single precursor was also published: ferrocyanide ions slowly dissociate ensuring a limited concentration of ferrous ions available for oxidation and, consequently, for PB formation. The synthesis was carried on using only a water solution of potassium ferrocyanide and hydrogen chloride as reagents [35]. Controlling the precursor concentration and the reaction temperature a good control on size and shape of the particles was achieved.

**Figure 4:**
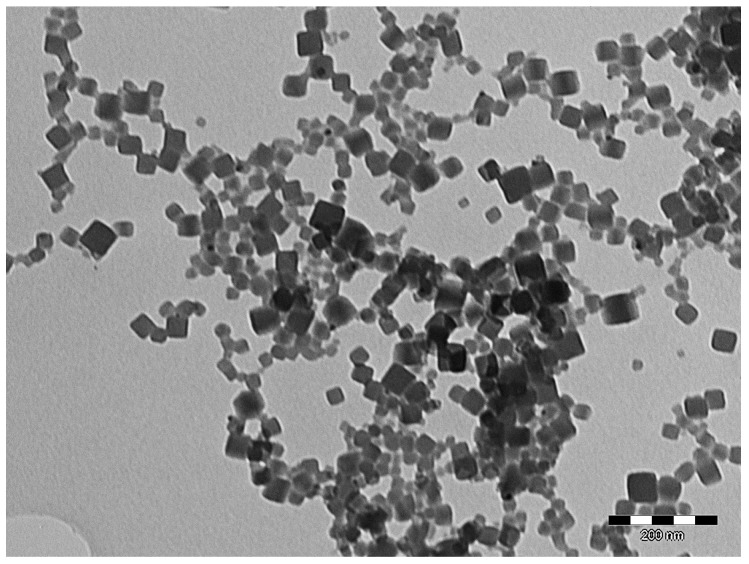
TEM micrograph of citrate-stabilized PBnp, prepared following Ref. [10] (authors’ unpublished data).

The most common route for the synthesis of PBnp in water without a polymeric o polyelectrolyte capping agent is the citrate/citric acid capped synthesis. It was initially believed that citric acid could act only as the reductant, and the use of a protecting polymeric agent was mantained in the synthesis (e.g., gelatin) [36]. The use of a polymer matrix or a protecting cage was then abandoned in favor of a synthesis involving simply a Fe^III^ salt, ferrocyanide and citric acid. This procedure yields single-crystal-like particles, typically with a cubic shape (see Figure 4), with promising features like an easy surface functionalization, a good solubility in water, alcohol and water-DMSO mixtures and the absence of cytotoxicity. The only drawback is a relatively wide size distribution [16]. Citrate capping thus seems to be the best compromise for a synthesis in mild conditions, with green reagents, yielding good quality shape-controlled nanoparticles.

### 2.2. Less Symmetric Shapes

Contrary to what happens with noble metal nanoparticles, literature reports only a few examples of anisotropic PBnp. Prussian blue is indeed more likely to assume the cubic form when synthesiszed in colloidal form. In some conditions, less regular, spheroidal particles are obtained [10,17]. Regarding less symmetric nanostructures, only a few examples are reported for the preparation of nanowires [37,38] and nanotubes [39]. Both nanowires and nanotubes are typically synthesised with a porous alumina template. Nanowires [38] were prepared by electrodeposition, while nanotubes were synthesised with a sequential deposition technique, tuning the length and the inner and outer diameters of the tube [39]. Both preparations are on the solid state, so no UV-Vis data is available for these nanostructures to be compared with symmetrical particles.

A different approach, i.e., the use of a natural surfactant, *Sapindus mukorossi* (raw ritha), was recently exploited for the green synthesis of sharp hexagonal PB nanorods (less than 60 nm in size) [40,41]. The synthetic protocol is based simply on the addition of a solution of K_4_[Fe(CN)_6_] to a solution containing Fe(NO_3_)_3_ and the plant extract. The synthesis yields insoluble hexagonal low aspect-ratio rods, that are used by the authors for their photocatalytic activity (a band gap of 1.145 eV was determined by means of reflectance measurements).

The complexity of obtaining non-symmetrical particles and their scarce solubility make them less interesting for the applications as photothermal objects. Moreover, even if no data is present in literature on their optical properties, it is reasonable to assume that the shape of the particle has little influence on the position of the absorption band of PB, contrary to what is observed with metal nanoparticles. In the latter case control of the shape of the particles is crucial to tune the position of the plasmonic resonance towards the bio-transparent near-IR region of the spectrum.

### 2.3. Hollow PBnp

Obtaining hollow nanostructures and nanocages with PB and its analogs is way easier than preparing anisotropic particles, as confirmed by the larger number of papers reporting this kind of structures. Wang et al. were the first to report these syntheses, with an approach called miniemulsion periphery polymerization (MEPP). MEPP consists in the preparation of miniemulsion droplets using organo-metallic surfactants, followed by polymerization at the periphery of the droplets. In a typical experiment, miniemulsion droplets with a pentacyanoferrate periphery were prepared from an organometallic surfactant of poly(ethylene glycol)-b-poly(propylene glycol)-b-poly(ethylene glycol) terminated with pentacyano(4-(dimethylamino)pyridine)ferrate (EPE-Fe) and a mixture of water, toluene and hexadecane was used as a solvent. Through this approach, spherical nanoshells [42], crystalline cubic nanoboxes [43] and tunable size amorphous solid nanocubes were prepared [44]. In the latter case the use of a different non-ferrate containing co-surfactant (together with the aforementioned EPE-FE) led to different particle size: ≈250 nm vs. <100 nm. The presence of the co-surfactant is also crucial for the sytnehsis of cubic nanostructures: in presence of pure EPE-Fe only spherical hollow PBnp are obtained.

In alternative to the microemulsion strategy, hollow PBnp can be prepared via controlled chemical (acidic) etching of solid PB nanocrystals [45]. PB nanocrystals obtained with this synthesis can also be converted to nanoporous iron oxides by calcination, yielding high surface area hematite crystals [46].

None of the cited papers reported a UV-Vis characterization, so, to our knowledge, no data is available in literature on the influence of a hollow structure on the PB absorption band.

The most interesting characteristic of HPBNP, however, is the possibility to load drugs inside the particle improving the loading capacity when compared to solid particles. This makes HPBNP ideal candidates for the preparation of theranostic devices. Typically, these devices combine the photothermal properties of prussian blue with imaging features and loading/release of chemotherapic drugs. This topic will be discussed more in detail in Section 5.

## 3. Nanoparticles and the Photothermal Effect

The so-called photo-thermal effect is the capability of a material (e.g., a magnetic or metallic particle) to convert an impinging electro-magnetic radiation into heat. In the case of metals, this is due to a (2–5 picoseconds) electron-phonon coupling with the metal lattice, followed by phonon-phonon relaxation on a longer timescale (100–400 picoseconds). For noble metals, the conversion of irradiation into heat is related to the absorption contribution of the plasmon resonance. Mie scattering, on the other hand, is responsible of the radiative dissipation [47]. Non radiative relaxation can take place via electron-electron, electron-lattice or electron-surface collisions. As mentioned before, the lattice cools at slower rates via phonon-phonon processes which lead to a local hyperthermia of the medium surrounding the nanoparticle. This has been widely exploited for the so-called photo-thermal therapy (PTT): i.e., the ablation of cancer cells through a local heating caused by laser irradiation of photothermally active nanoparticles. For PTT the wavelength of irradiation plays a crucial role: for a usability in vivo the nanoparticles must have a maximum absorption wavelength in the bio-transparent region of the spectrum (i.e., in the near-IR; 750–1100 nm). Metal nanoparticles have been extensively studied in literature for this purpose, with a particular attention devoted to non-symmetrical nanoparticles. The position of LSPR bands of metals can in fact be tuned in the spectrum just by changing the aspect ratio of the objects. Gold nanoparticles have been used in several shapes: e.g., nanorods [48], nanostars [49,50,51], nanocages [52] just to cite the most common. Silver has similar characteristics, and Ag nanorods [53] and nanoprisms [54,55] are the preferred shapes that lead to a good LSPR tunability.

In order to understand the phenomenon of the photothermal effect and to better exploit it for PTT, studies were published on the heating of a fluid medium in presence of photothermally active nanoparticles. In a seminal paper Keblinski et al. [56] showed that, even if a single particle generates heat in a higly localized volume, the temperature field is essentially homogeneous over the entire volume of the medium exposed to irradiation. However, when heating is caused either by radio frequency (rf) magnetic fields applied on magnetic nanoparticles or on continuous wave lasers irradiating metallic (gold) photothermal nanospheres, the local temperature rise that is ascribable to a single particle is almost negligible. It is the overall, large number of nanoparticles that generates a global temperature rise, that is orders of magnitude larger than the temperature increase adjacent to a single particle. In order to obtain a localized heating larger than the bulk heating, high power optical pulses with a low duty cycle are needed. Vo Dinh and Norton [57] recently published a similar study, extending the conclusions of Keblinski and coworkers by analyzing the different behaviour of spherical and non-spherical (i.e., nanospheroids, nanostars) gold nanoparticles under laser irradiation. This is relevant for a possible use in vivo of such nanostructures, since non symmetrical gold nanoparticles have plasmon resonances falling in the bio-transparent region. Nanocages were not considered in this study, because they have a lower cross-section if compared to solid structures. Treating the nanoparticles as point sources and assuming they are confined in a limited region, the steady-state temperature of the fluid medium can be calculated and the time constant to reach this temperature can be determined too.

## 4. Tuning the PBnp Maximum Absorption Wavelength

The typical blue color of PB is due to a charge transfer (CT) transition, i.e., to the transfer of one electron from the Fe^II^ centers to the Fe^III^ centers. PB is a Class II mixed valence compound, according to the Robin and Day classification [6]. The maximum absorption wavelength of CT bands involving metal centres are strongly influenced by changes in the coordination sphere and by the environment (solvent) dielectric constant [58]. As it has been already mentioned in the introduction, values of λ_max_ ‘around 700 nm’ are typically reported in the literature for PB solutions, with a variability that is reasonably due to changes in the coordination sphere of the Fe^3+^ cation. This ranges from 6 N atoms (of the cyanide anions) in “soluble” PB to an average of 4.5 N atoms plus 1.5 oxygen atoms (H_2_O) in the ‘insoluble’ PB. A spectroelectrochemical study from Mortimer and Rosseinsky clarified the difference between the spectra of the “insoluble” and “soluble” forms of PB. The first has a maximum absorption wavelength of 730 nm and a larger FWHM, while the latter absorbs at 690 nm with a narrower band [59]. It has to be stressed that these values are measured on thin solid films deposited on the surface of an electrode. A similar preparation of ‘insoluble’ PB, Fe^III^_4_[Fe^II^(CN)_6_]_3_, deposited on a SnO_2_ electrode, lead to the determination of λ_max_ = 700 nm [9]. The molar extinction coefficient calculated in the same paper agrees with the CT nature of the PB absorption band, being 9800 L mol^−1^ cm^−1^ with respect to Fe^2+^ and 7300 L mol^−1^ cm^−1^ with respect to Fe^3+^. On the other hand, it must be stressed that the typical solvatochromism of the CT bands is only a marginally observed in PBnp, due to the continuous nature of this material. Although in a nanoparticle the surface atoms are in a not negligible number with respect to the bulk ones, the large majority of Fe^III^ and Fe^II^ centres are in the core of the PBnp and not influcenced by solvent. A simple calculation can be carried out using the data of Ref. [3]. A cubic primitive elementary cell with parameter a = 1.0166 nm was found by X-ray diffraction for ‘insoluble’ PB, each cell containing 4 Fe^3+^ and 3 Fe^2+^ cations. Using these parameters, a PBnp nanocube of 50 nm side contains ~1.19 × 10^5^ elementary cells, with 4.76 × 10^5^ Fe^3+^ and 3.57 × 10^5^ Fe^2+^ cations. A shell with the thickness of one elementary cell on the surface of a 50 nm PBnp contains 2419 × 6 = 1.45 × 10^4^ elementary cells, i.e., 5.80 × 10^4^ Fe^3+^ and 4.35 × 10^4^ Fe^2+^ cations, corresponding to 12% of the total Fe atoms. Uemura and coworkers studied the different positions of PBnp absorption dissolving PVP-protected PBnp in different organic solvents [27]. Data were compared with the absorption of the same nanoparticles in water, which is centered at 689 nm (i.e., blue-shifted with respect to the absorption of a “bulk”, non protected, PB in water, λ_max_ centered at 700 nm). The position of the absorption band does not show a sharp dependence on the polarity of the solvent. The authors propose an explanation based on the different solubility of the PVP-protected PBnp in the different solvents: in good solvents such as water, MeOH and DMSO, the solvent permeate easily in the polymeric layer, weakening the interaction of PVP with the inorganic nanoparticle. On the other hand, the PVP interaction is stronger for solvents in which PBnp are poorly soluble, thus inducing a greater blue shift of the band (λ_max_ = 672 nm in CHCl_3_).

The aforementioned difference in the position of the CT band of soluble and insoluble PB corresponds also to the presence/absence of the “supernumerary” K^+^ cations, needed to mantain electroneutrality in ‘soluble’ PB. It has been observed that the substitution of K^+^ with other cations can further tune the optical properties of PB. With alkali metal ions, the CT λ_max_ shifts are proportional to the PB mass changes on M^+^ insertion [60]. Rosseinsky and coworkers put this in relationship with the progressively weaker interaction of the bigger cations with CN^-^. In the case of M^2+^ and Ag^+^ ions, the shifts observed in λ_max_ are again put in relation with the interactions of the supernumerary ions with the fixed lattice ions, via the CN^-^ of the Fe^II^(CN)_6_. The authors proposed that those interactions are well represented by the solubility products of the metal ferrocyanide salts. Among all the metal ions that can be included in the PB lattice, Mn^2+^ raised a particular interest in the field of photothermal therapy. In the presence of Mn^2+^ as “doping” ions the absorption band of PBnp can be red-shifted up to 768 nm, fully entering the biotransparent window. It has to be stressed that in this case Mn^2+^ are not “supernumerary” ions, but they are part of the PBnp lattice, replacing Fe^3+^ ions, as demonstrated by means of X-ray diffraction measurements [61]. Such a consistent red-shift in the band, without losing its intensity, can lead to an important improvement of the photothermal efficiency in vivo, where the choice of the irradiation wavelength is limited to the bio-transparent region of the spectrum.

In conclusion, there are several factors which can influence the position of the absorption band of PBnp: the solvent, the capping agent surrounding the particle, the presence and the nature of “supernumerary” ions. A thorough explanation of the nature of all these effects has still to be found, but what we can say is that tha influence of all the aforementioned factors on the position of the band is small, and not of particular relevance for a photothermal application. The most promising way to obtain a significative red-shift of the band is the use of doping ions, provided that the substitution of Fe ions with other metals does not reduce the molar absorption.

## 5. Photothermal Properties of PBnp and Their Biomedical Use

To our knowledge, the first study reported in literature regarding the photothermal effect of PBnp was published by Fu et al. in 2012 [7]. In this paper, citric acid capped PBnp were studied for their photothermal properties in comparison with a traditional nanostructured photothermal agent, i.e., CTAB-capped gold nanorods. PBnp showed a lower molar extinction coefficient (if compared to gold nanorods), but better photothermal stability. Most importantly, PBnp proved to be an efficient PTT tool for the treatment of HeLa cancer cells, showing no cytotoxicity in absence of laser irradiation and minimum cell viability upon irradiation. Similar results were obtained by Li et al. [62] with hepatocellular carcinoma. The authors also combined the PTT effect with a study on MR imaging in vitro, using antibody functionalized PBnp as theranostic agents.

**Figure 5:**
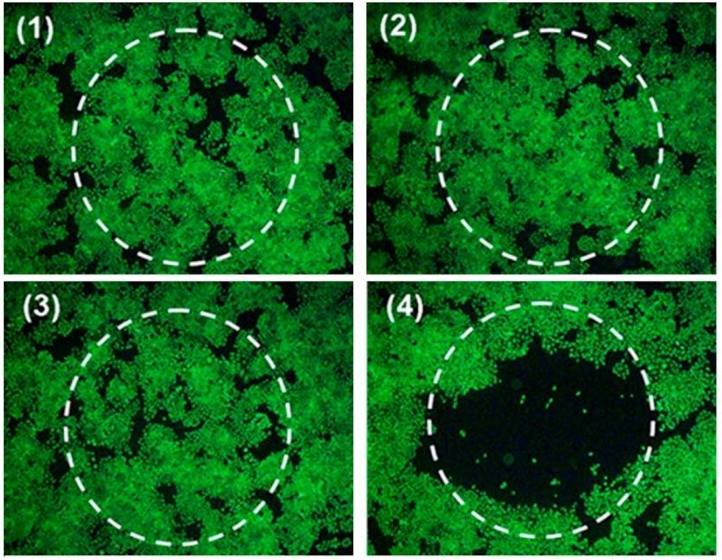
Adapted with permission from Ref. [64]. Photothermal destruction of Hela cells with or without Au@PB NPs and NIR laser (808 nm, 1.5 W·cm^−2^) treatments.

The first examples of in vivo studies on the photothermal effect of PBnp were published in 2014. Hoffman and coworkers [63] reported the effect of PBnp against neuroblastoma in mice, with promising results in terms of tumor debulking, increase in tumor-free days and decreased tumor growth rates. Similar results in terms of tumour photothermal ablation in vivo were obtained by Jing et al. [64], who realized a theranostic nanostructure based on a spherical gold nanoparticle core (9 mm in diameter) serving as a CT contrast agent, coated with a PB shell acting both as the PTT agent and as a contrast agent for photoacoustic imaging (in vitro photothermal effect of these nanostructures on HeLa cells is shown in Figure 5). PB-based core-shell nanostructures were realized also coating a superparamagnetic Fe_3_O_4_ core with a PB shell [65]. In vitro and in vivo studies on these nanodevices showed significant contrast in MRI imaging, togheter with a high photothermal effect under irradiation in the near IR, thanks to the absorption of the PB shell in this region. Tumor ablation and tumor growth inhibition properties were confirmed both on HeLa cells and with in vivo studies on tumor bearing nude mice.

From these few seminal papers the potential of PBnp for biomedical use is already evident: in the last years (i.e., from 2014 to 2017), around 50 papers have been published regarding the photothermal effect of PBnp. Imaging-guided laser ablation is indeed a minimally invasive technique, which can be used as a therapeutic approach for cancer. PBnp proved effective in the treatment of some of the most common tumour cells: neuroblastoma, hepatocellular carinoma, HeLa cells. Moreover, studies in vivo showed promising results and the biocompatiblity of PB is well known.

Many of the papers describing the photothermal effect of PBnp propose a combined use of the photothermal properties coupled with imaging, drug delivery and chemotherapy functions. The next paragraphs will review the most common imaging techniques use in combination with PTT and some of the synergistic therapeutic approaches.

### 5.1. Magnetic PBnp

The aforementioned paper by Li et al. [62] was the first to envisage the possibility of using PBnp as MRI contrast agents, since an excellent MRI contrast-enhancing ability was obtained with antibody functionalized PBnp.

In the last years, several studies have been published on PBnp (both pure prussian blue and hybrid structures), capable of combining photothermal effect and magnetic properties, the latter being exploitable for MRI imaging.

One of the most used synthetic strategies to realize theranostic devices for MRI imaging and PTT is the combination of superparamagnetic iron oxide nanoparticles (SPIONs) with prussian blue. Core-shell structures made of a SPION core and a prussian blue shells have been studied in several papers [65,66,67]. Prussian blue shells exert a good photothermal effect (comparable to the one observed for homogeneous PB nanostructures) since their solutions can reach temperatures higher than the critical value of 43 °C, required for tumor cells ablation. Concentrations around 100 µg/mL and 10 min of irradiation (4 mL samples were irradiated with a 2 W continuous-wave laser, irradiance was not reported in the paper) are typically required to reach the critical tempearture [66,67]. An interesting feature of magnetic PBnp is the presence of a magnetic field enhanced photothermal effect: the presence of an external magnetic field during laser irradiation enhanced considerably the ablation of HeLa cells in in vitro tests [66,67]. This has been explained with a magnetic targeting effect, i.e., the capability fo the magnetic field to draw the nanoparticles towards the bottom of the cell culture, thus improving cell internalization [65]. The photothermal effect of magnetic PBnp, togheter with their MRI contrast agent properties have been confirmed also in vivo on U87MG tumor bearing nude mice. In this case, mice were irradiated with a 1.5 W·cm^−2^ irradiance for 10 min, after the injection of 0.1 mL aqueous or saline suspensions (1.0 mg·mL^−1^) of the Fe_3_O_4_@PBnp [65].

Lanthanides are a good alternative to iron oxides as MRI contrast agents. Mixed dysprosium and lanthanium nanoparticles have been used to prepare core/shell structures, in combination with prussian blue [68]. Lanthanium-doped NaDyF4 were synthesized by solvothermal method and coated with a PB shell. Good performances were recorded both in vitro and in vivo for these materials, but their biocompatibility, especially regarding the mechanism of excretion of lanthanides based nanoparticles, is still to be studied in detail.

Another strategy for enhancing photothermally active PBnp with magnetic properties is the doping of the nanoparticles with the well-known MRI positive contrast agent gadolinium [69]. potassium/gadolinium hexacyanoferrate is simply synthesized mixing a Gd^3+^ salt with Fe^3+^ chloride, thus partially substituting iron(III) ions with gadolinium. Reported nanostructure have an increasing gradient of Gd ions going from the core towards the surface of the particle, thus maximizing the efficiency of the MRI active metal (which must be in contact with water to be effective). Gd-doped PBnp are non toxic and maintain the photothermal properties fo non-doped PBnp.

**Figure 6:**
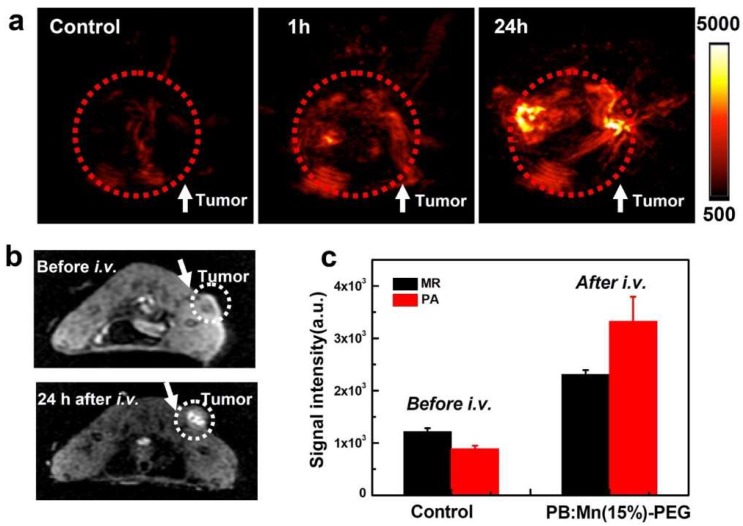
In vivo photoacoustic and MR imaging with Mn^2+^ doped PBnp. (**a**) 2D photoacoustic imaging of tumors before and after injection of Mn^2+^ doped PBnp. (**b**) In vivo T1-weighted MR images of a mouse taken before injection (**upper**) and 24 h post injection (**bottom**) of Mn^2+^ doped PBnp. Brightening effect showed up in the tumor after i.v. injection of Mn^2+^ doped PBnp. (**c**) T1-weighted MR signal intensities and PA signal intensities in the tumor before injection and 24 h post injection of Mn^2+^ doped PBnp. Reprinted with permission from Ref. [60]. Copyright 2015 American Chemical Society.

Mn^2+^ ions were also used for PBnp doping, obtaining interesting results in terms of high longitudinal relaxivity, which makes these nanoparticles promising MRI contrast agents (Figure 6 shows the results obtained for MRI and PA imaging in vivo with this nanoparticles, with the relative enhancement effects), and photothermal properties (in vitro and in vivo), thanks to a red-shift of the maximum absorption wavelength when PB is doped with Mn^2+^ ions [60]. Mn(II) doping of PB can induce a red-shift of the metal-to-metal charge transfer of up to 64 nm (from 702 nm to 768 nm, with a 25% Mn(II) doping referred to the total Fe).

Interestingly, also the PBnp as such could be used as MRI contrast agent, without the need of a hybrid nanomaterial [16,62]. This has been described by Dou and coworkers (in combination with Au nanoparticles as CT contrast agents and photothermal effect enhancers) [70] in order to realize a PBNP based theranostic device. Cellular tests on HeLa cells showed that the PBnp have a clear MRI signal brightening effect, togheter with the well-known photothermal effect. The use of a pure PBnp as contrast agent without any doping or core-shell materials is very promising, since it does not introduce any potentially harmful or cytotoxic chemical in the nanostructure. It has however to be stressed that PB has a longitudinal relaxivity value one order of magnitude lower than Gd based contrast agents approved for clinical use [71].

### 5.2. PBnp for PTT and Photoacoustic Imaging

Photoacoustic (PA) imaging is a diagnostic technique based on the use of non-ionizing laser pulses inducing local heating, a transient thermoelastic expansion and a consequent wideband ultrasonic emission. PBnp, with their strong absorption band in the NIR are ideal candidates as PA imaging contrast agents. Liang et al. [72] were the first to report the use of PBnp as PA imaging contrast agents. The photoacoustic efficiency of PBnp were found to be greater than that of hemoglobin in blood. PBnp were proved to be effective both in vitro, with good depths of penetration of the 765 nm laser pulse trough tissues, and in vivo after intravenous administration.

**Figure 7:**
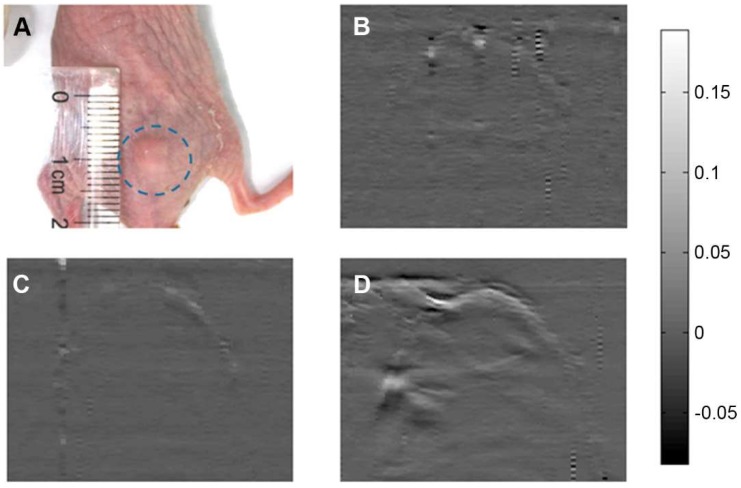
Photoacoustic imaging in vivo with PB coated Au nanoparticles as contrast agents. (**A**) The nude mice tumour before the acquisition. PAT images aquired (**B**) before, (**C**) 2 h after, and (**D**) 22 h after the tail intravenous injection (reproduced with permission from Reference [64]).

The aforementioned work from Jing and colleagues [64] exploited the properties of core-shell Au@PB nanostructures as a theranostic agent, combining PA imaging with the photothermal effect. While the Au core was exploited as CT contrast agent, the PB shell and its NIR absorption was used for both the PA imaging (as shown in Figure 7) and the photothermal effect: the broad absorption band centered at 700 nm grants indeed a good a reponse for both the used laser wavelengths (i.e., 765 nm pulsed laser for PA imaging, 808 nm continuous-wave laser for PTT).

Besides solid PBnp nanoparticles and core-shell nanostructures, hollow PBnp and doped PBnp have also been used, with good results, as combined photothermal therapy and PA tomography agents. The use of hollow mesoporous PBnp adds the possiblity to load a drug inside the particles, thus obtaining a synergistic “chemo-thermal” tumor therapy [73]. Doping the PBnp, on the other hand, has an effect on the position of the absorption band: this allows to improve the efficiency of the PA and PTT effects, thanks to a red-shift of the maximum absorption wavelength (i.e., a shift towards the bio-transparent region of the spectrum) [74]. It has to be stressed that also the doped-PBnp can be obtained as hollow mesoporous nanostructures.

### 5.3. Gene Delivery and Photothermal Therapy

Nanoparticles made of several different materials have been used as carriers for therapeutic genes in gene therapy (i.e., the therapeutic delivery of genes used as drugs). Nanoparticles are good vectors for gene therapy thanks to their enhanced permeation and retention in tumors, and are considered promising alternatives to the viral gene-delivery carriers [75]. Gene therapy and photothermal ablation have been applied in synergy in some recent studies: gold nanoparticles covalently linked to a viral vector [76] PEG- and PEI- modified graphene/Au composites [77], Au nanorods [78], single-wall carbon nanotubes (SWNT) [79] have been proposed as gene delivery nanosystems with a photothermal effect. In these systems the photothermal effect acts with its typical ablation process, but it can also serve as a trigger for gene delivery, because a mild photothermal effect (<43 °C) can increase the fluidity of the cell membrane. In addition to this, the temperature increase can promote the drug release from the carrier [75].

Li et al. [80] proposed the use of chitosan-coated prussian blue nanoparticles as photo-controllable gene delivery devices. PBnp are ultra small (3 nm) and positively charged. Upon irradiation with NIR light, chitosan-coated PBnp showed an improved gene transfection efficiency, alongside with a great biocompatiblity and stability in the biological environment. The same authors proposed also a more complex system, adsorbing the chitosan-coated PBnp onto the surface of gas encapsulated microbubbles [81]. This approach allows to join the previously described photothermally-enhanced gene deliery with ultrasound-targeted bubble distruction. Ultrasounds are used both for imaging and for the targeted distruction of the microbubbles, which improves the efficiency. The combined used of ultrasound irradiation and NIR irradiation enhanced the gene transfection efficiency significantly, up to 43%.

Enhanced gene transfection was also studied by Xue and coworkers in combinations with magnetic properties: chitosan-coated PB@Fe_3_O_4_ core-shell nanoparticles were exploited as gene carriers [67]. The presence of a superparamagnetic core allowed efficient magnetic targeting on HeLa cells.

### 5.4. Photothermal Chemotherapy

Besides the innovative gene-delivery therapy, PBnp were also studied as drug carriers with more traditional chemotherapeutic drugs, e.g., doxorubicin. The first example in this field is rather recent: in 2015 Xue et al. prepared Prussian Blue nanoparticles coated with gelatin-doxorubicin conjugate [82]. Drug release was triggered by gelatinase, an endogenous proteolytic enzyme overexpressed in tumour tissues. These materials were tested against HuH7 tumour cells in vitro, showing a good photothermal effect and a synergistic effect between phothermal therapy and chemotherapy. The same authors [66] reported a theranostic device based on Fe_3_O_4_@PB core shell-nanoparticles coated with gelatin-doxorubicin conjugate (Fe_3_O_4_@PB@GEL-DOX NP). The three main components of this system (i.e., PB, DOX, Fe_3_O_4_) are all FDA approved and the Fe_3_O_4_@PB@GEL-DOX were succesfully synthesised combining photothermal properties, magentic properties and the possibility of loading and delivering a typical chemotherapeutic drug.

A similar system was realized loading doxorubicin in PB nanocages [83]. Doxorubicin release studies at different pH values and in presence/absence of laser irradiation showed that local hyperthermia can enhance the drug release, leading to a significant enhancement of the therapeutic effect against hepatocellular carcinoma cells in vitro. A similar theranostic device (i.e., hollow mesoporous prussian blue nanoparticles, HMPBNP, loaded with doxorubicin) [73] was tested for its in vivo biocompatibility, confirming excellent pharmacokynetics and negligible long-term toxicity. Cell viability was tested with HeLa, 4T1 and PANC1 cells, showing no cytotoxicity of the HMPBNP and the synergistic effect between photothermal therapy and chemotherapy was verified with in vivo tests on tumour bearing mice. Results confirm that an increase in temperature weakens the electrostatic and coordinative bond between DOX and Prussian Blue, enhancing the drug release upon irradiation. Doxorubicin release is also triggered by a variation in pH, offering a dual-mode on-demand release upon irradiation or a pH change (typical of the tumour cell environment) [84].

Another delivery system was realized exploiting a phase change material (PCM): Chen and coworkers [85] prepared hollow mesoporous PBNP loaded with doxorubicin and 1-tetradecanol (which has a melting point around 38 °C). The PCM molecule acts both a as a thermosensitive delivery medium and as a solvent for both hydrophilic and hydrophobic anti-cancer drugs.

In addition to the widely used doxorubicin, other chemotherapeutic drugs have been studied in combination with PBNP. MEK inhibitors (i.e., PD-0325901) were used to treat malignant peripheral nerve sheath tumours [86]. 10-hydroxycamptothecin was studied in combination with hollow Prussian blue nanoparticles modifed with hyaluronic acid grafting polyethylene glycol [87]: in this case the hyaluronic acid serves as targeting moiety for the CD44 receptor, which is overexpressed on HeLa cells, while 10-hydroxycamptothecin is a chemotherapeutic drug that showed good activities against gastric carcinoma, hepatoma, leukemia and tumour of the head and neck. In vitro and in vivo studies confirmed the targeting properties of this nanodevices, their photothermal activity and the light triggered drug release.

### 5.5. Photothermal Antibacterial Effect

As we mentioned at the beginning of paragraph 5, the study of the photothermal effect of PBnp for biomedical use is a recent research topic. An even more recent one is the employment of PBnp and their analogs as photothermal antimicrobial agents. Anisotropic metal nanoparticles (i.e., the ones that can be irradiated in the bio-transparent near-IR region of the spectrum) are well-known switchable antibacterial agents. Several examples have been reported on the use of metal nanoparticles for photothermal killing of pathogenic bacteria [88,89]. Metal nanoparticles were used for the formation of Self-Assembled Monolayers, to prevent the formation of biofilms [90].

To our knowledge, only two papers can be found in literature on the use of PBnp as photothermal antibacterial agents. The first one is the work of Maaoui and colleagues [91] on the photothermal ablation of Gram+ and Gram- bacteria using PVP coated PBnp. Photothermal effect was tested at 810 nm and 980 nm at 1 W·cm^−2^ irradiance. By choosing an appropriate PBnp concentration, a good selectivity in the killing of bacteria over mammalian cells (HeLa) was achieved. The second paper was published in 2017 by our group [10], and focuses on the antibacterial photothermal effect of Self-Assembled Monolayers (SAM) of PBnp on glass. PBnp were grafted on a glass surface via a layer-by-layer approach: the substrate is functionalized with a polyamine bearing silane groups, capable of forming covalent Si-O-Si bonds with the glass surface. The obtained SAM is then used to firmly graft a layer of photothermally-active PBnp, capable of exerting an antibacterial activity against Gram− (*E. coli*) and Gram+ (*S. aureus*).

## 6. Patented Applications of Photothermal PBnp

In the first two decades of the 21st century, more than 2000 patents are deposited each year regarding “Prussian Blue” (as a research query on Scopus). The query “Prussian Blue nanoparticles” yields only 40 patent results, with diverse applications: electrochemistry, electrochromic materials, sensing, water and soil purification, contrast agents, just to cite a few.

The use of PBnp as photothermal theranostic devices is a very recent topic. Only a few patents were deposited on the preparation and use of PBnp in biological environments exploiting the photothermal effect, and most of the patents are strictly related to scientific literature. For example, the first patent on the use of PBnp for diagnosis and treatment of cancer [92] was deposited by Dai and Fu, authors of several papers mentioned in this review [7,64,65,72,80,81,87]. In this patent, core-shell nanostructures made of iron oxide core and PB shell are proposed as theranostic devices for magnetic resonance imaging and photothermal therapy for cancer. Another patent on the combined use of PBnp for MRI and photothermal therapy was deposited by Liu et al. [93], describing a material similar to what they reported in Reference [61]: antibody-functionalized PBnp reported in this patent have a good target recognition function towards hepatocellular carcinoma cells and show an effective internalization. Meanwhile, they have an excellent photothermal killing function against the same cells and can be used for photothermal therapy. This antibody-functionalized PBNPs have a good nuclear magnetic resonance contrast imaging function and have a potential of becoming a new generation of a target magnetic resonance imaging contrast agent.

A photothermal-chemotherapy combined therapeutic agent based on PB was patented by Wang et al. [94], following the same synthetic approach they described in References [42,43,44]. The therapeutic agent is composed by a PBnp as a core and a drug-carrying layer is formed outside the core through hydrophobic-hydrophobic interaction between two different amphipathic surfactants.

Fernandes and coworkers [95] reported a combined approach with photothermally active PBnp or PB analog nanoparticles and immunotherapic treatments. Immunotherapies include the use of antigen-specific T cells conjugated to PBnp to target antigens of tumors and microbial pathogens, the use of inhibitors, such as MEK inhibitors (as seen in Reference [86]) or checkpoint inhibitors in combination with Prussian blue photothermal therapy for treatment of neuroblastoma or other types of cancers, tumors or malignancies.

These recent patents describing innovative theranostic approaches reveal an increasing interest towards the clinical applications of PBnp and their derivatives and analogs.

## 7. Conclusions and Future Outlook

Prussian Blue in its colloidal form is a non-toxic, versatile material easy to synthetize in mild conditions. The first synthesis of PBnp are two decades old, and in the last years a wide interest has spread on the biomedical use of PB. The material is FDA approved as an antidote for Cs^+^ and Tl^+^ poisoning, and this makes PB an ideal candidate for biomedical purposes.

PBnp have interesting magnetic, optical and chemical properties and they can be used as imaging contrast agents and as carriers for drugs. Several studies cited in this review showed promising results in vitro and in vivo with different imaging techniques (e.g., photoacousitc imaging, MRI) and an efficient photothermal effect, also in combination with other therapeutic approaches (chemotherapy, gene therapy). Even if a good number of studies has been published, in the last years, it has to be stressed that some aspects of the synthesis and biomedical use of PBnp still needs a rationalization: for example the use of different stabilizing agents and coating molecules should has still to be explored, since most of the synthesis reported in literature are based only on citrate-capped and PVP-capped PBnp. The use of different capping agents can improve the stability in vivo (which was already proved to be good) and the presence of targeting moieties is useful to increase the active targeting of the photothermally-active particles in the tissues of interest.

Regarding the bio-stability and toxicity of PBnp, the fact that PB is already FDA approved (as the commercial product Radiogardase^®^) is an enormous advantage compared to other typical PTT nanomaterials like metal nanoparticles. A complete study of the biocompatiblity of PBnp in all its form (i.e., different size, shape, coating, etc.) however is still needed to ensure that PBnp has no negative effects, together with a complete study on the intracellular processes and on its accumulation and clearance in the biological environment.
